# The influence of psychological readiness of athletes when returning to sport after injury

**DOI:** 10.17159/2078-516X/2024/v36i1a16356

**Published:** 2024-02-15

**Authors:** C Juggath, R Naidoo

**Affiliations:** Discipline of Biokinetics, Exercise and Leisure Sciences, School of Health Sciences, University of KwaZulu-Natal, South Africa

**Keywords:** fear avoidance, athlete confidence, psychological assessment tools

## Abstract

**Background:**

Injuries are a common occurrence in sports participation; however, they have the potential to be accompanied by negative thoughts and feelings, which may play a part in the athletes’ state of mind when they return to their sport. Assessing the degree to which this occurs provides an opportunity to evaluate and address athletes’ state of mind before their return to play.

**Objectives:**

To determine if athletes are psychologically ready to return to play after an injury and if there are differences in fear avoidance behaviour between those who were and were not ready to return.

**Methods:**

Eighty-eight athletes participated in this descriptive survey. Athletes’ confidence to return to play was measured by the Injury-Psychological Readiness to Return to Play (I-PRRS) questionnaire and their fear avoidance was measured by the Athlete Fear Avoidance Questionnaire (AFAQ).

**Results:**

Fifty injured athletes with a mean age of 23.3±4.0 years old responded to the I-PRRS and the AFAQ questionnaires. The average I-PRRS score was 46.5±9.1 AU. The evidence suggests that 60% of the athletes were not ready to return to sport (41.0±7.5 AU), whereas 40% were ready to return (54.8±3.1 AU). The difference in scores was not significant. The relationship between the AFAQ scores and the I-PRRS score for the ‘ready’ and ‘not ready’ groups was not significant (p=0.066). The mean AFAQ score (26.1±8.6 AU) for the ‘not ready’ group is marginally greater than the mean AFAQ score (21.6±7.5 AU) for the ‘ready’ group. There was a negative correlation between psychological readiness to return to sport and athletic fear avoidance (r =−0.508, p<0.001).

**Conclusion:**

There needs to be a greater utilisation of psychological assessment tools like the Injury-Psychological Readiness to Return to Play (I-PRRS) questionnaire, which can assist the athlete’s support team, who can help identify athletes who are apprehensive about returning to sport after injury.

Sports can break down barriers and unite people of various backgrounds with a shared interest. The popularity of different and diverse sports provides entertainment for their respective fans, creates career opportunities, boosts the economy, and promotes social inclusion. ^[1]^

A key part of enjoying sports is observing athletes' skills, motivation, and perseverance. Although the public only witnesses the flawless execution of an athlete's skills, several underlying factors enable them to perform at their peak level. Furthermore, there are just as many obstacles that they need to overcome to be at their best. ^[2]^

As the number of people participating in sports has increased, so too has the number of injuries. ^[3]^ An injury, particularly a time-limiting or season or career-ending, may be a significant source of stress to athletes. ^[4]^ Athletes devote much time and energy to their sport. When athletes sustain injuries, it can be a traumatic experience that unexpectedly robs them of their ability to participate in their sport. ^[5]^ Therefore, athletic injury is associated with negative psychological responses. ^[6]^ Since injuries appear to be increasing in frequency, the potential negative emotional effects of athletic injury have become an increasing concern. ^[7]^ However, research on sports injuries has primarily focused on physical factors. ^[8]^ This is despite previous research indicating that sports injuries have psychosocial effects on athletes. ^[9, 10]^ Therefore, this study aimed to determine the influence of the psychological readiness of athletes when returning to sport after injury.

## Methods

### Study design and participants

The study was conducted using a descriptive survey design on athletes between 18 and 30 years old from various sporting teams and clubs in KwaZulu-Natal. A purposive sample of 88 athletes volunteered to participate in this study. Athletes who had any concussion-related injuries were excluded from the study. The 50 athletes who were injured filled in the two questionnaires: the Injury-Psychological Readiness to Return to Sport (I-PRRS) questionnaire and the Athlete Fear Avoidance Questionnaire (AFAQ) (Supplementary file).

### Ethical clearance

Ethical clearance was obtained from the Humanities and Social Sciences Research Ethics Committee (HSSREC) (Protocol Reference: HSSREC/00003002/2021). All participants signed an informed consent form before their involvement in this study.

### Data collection tools

#### Injury-Psychological Readiness to Return to Sport (I-PRRS)

The Injury-Psychological Readiness to Return to Sport (I-PRRS) questionnaire consisted of six items which assess overall confidence to play, confidence to play without pain, confidence to give 100% effort, confidence to not concentrate on the injury, confidence in the injured body part to handle the demands of the situation and confidence in skill level/ability. The response scale for each item ranged from 0 to 10, where a score of 0 implied that the athlete had little to no confidence, a score of 5 implied moderate confidence, and a score of 10 implied that the athlete had utmost confidence for that item. ^[11]^ The maximum score was 60. A score of 60 implied that an athlete had the utmost confidence to return to sport at that time; for a score of 40 the athlete had only moderate confidence; and for a score of 20, the athlete had low overall confidence. ^[11]^ For the purpose of this study, athletes who scored less than 50 on the questionnaire were regarded as ‘not ready’. The data are reported as arbitrary units (AU).

#### Athlete Fear Avoidance Questionnaire (AFAQ)

The Athlete Fear Avoidance Questionnaire (AFAQ) is a questionnaire that helped to establish how pain-related fear can influence the athletic population, specifically regarding rehabilitation. ^[12]^ The AFAQ comprised ten items which are rated on a scale from 1 (no fear avoidance) to 5 (high fear avoidance). The maximum score is 50, which implies a high degree of fear avoidance behaviour while the lowest score is 10, which implies little to no fear avoidance behaviour. ^[12]^ The data are reported as arbitrary units (AU). Both questionnaires are in the supplementary file.

### Statistical analysis

Descriptive statistics were used to report means and standard deviations. Independent sample t-tests were conducted to determine if AFAQ scores differed between the ‘ready’ and ‘not ready’ groups. The Mann-Whitney test was also conducted due to small deviations from normality within the AFAQ scores. The results were the same as the results of the t-test. To determine the correlation between athlete fear and readiness to return to sport, we used Pearson's correlation coefficient. Significance was set at p<0.05.

## Results

### Participants’ characteristics

The sample comprised 50 athletes who were injured. The mean age was 23.3±4.0 years old, with 70% being male, 26% female and 4% not specifying their gender.

The average number of days per week these athletes spent training was 5.1±1.1 days, and the average number of hours trained per day was 2.80±1.52 hours. Athletes dedicated most days to strength/flexibility training (4.1±1.9 days) and cardiorespiratory fitness (4.1±2.0 days), with the least number of days being dedicated to power training (2.1±1.3 days) and unspecified training (0.3±0.6 hours).

A large majority of the athletes were out of play due to injury for one or two weeks (10% and 17% respectively), while only one athlete was out of play for 32 weeks (1%) and one who was out of play for 36 weeks (1%). The most common type of injury was muscle strains (16%) while the most common location of injuries was the knee (11%).

### I-PRRS scores

**Fig. 1:**
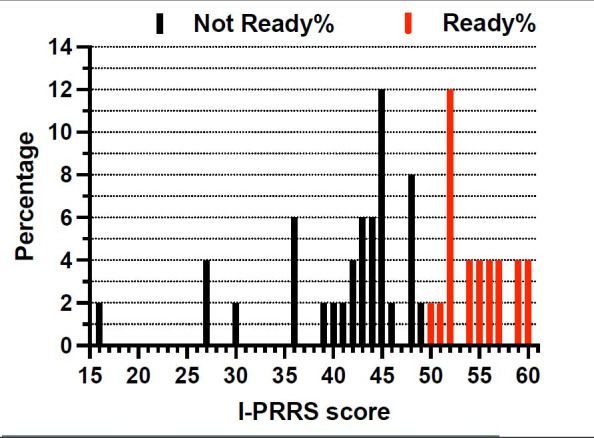
Distribution of Injury-Psychological Readiness to Return to Sport (I-PRRS) scores. Readiness to return to sport is expressed as a percentage (%). An I-PRRS greater than and including 50% is deemed “ready” (red)

The most common scores were 45 and 52, accounting for 7% of the scores. According to their scores, 30 athletes were grouped as ‘not ready’ (60%), and 20 athletes were grouped as ‘ready’ (40%). Figure 1 shows the percentages of the distribution of the scores on the I-PRRS questionnaire amongst the participants.

### AFAQ scores

**Fig. 2:**
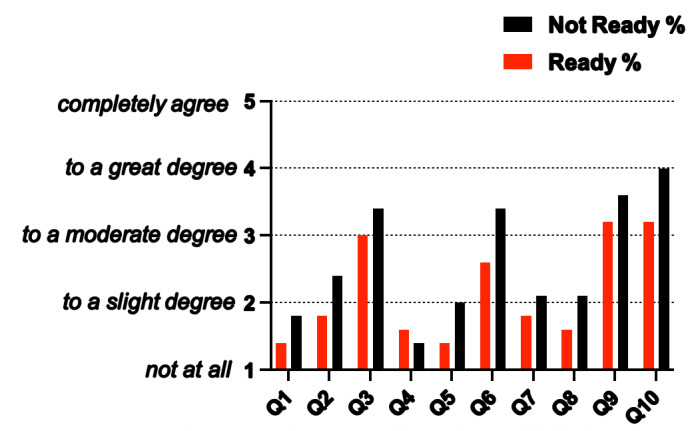
Differences in mean scores for each Athlete Fear Avoidance Questionnaire (AFAQ) question between the two groups (“ready to return” and “not ready to return”)

In the AFAQ scores between the ‘ready’ and ‘not ready’ groups, the results were not significant (p=0.066). Mean AFAQ scores for the ‘not ready’ group (26.1±7.5 AU) are marginally greater than mean AFAQ scores for the ‘ready’ group (21.6±8.6 AU) but this was not significant (Figure 2).

### Correlation between psychological readiness to return to sport and athlete fear avoidance

There was a negative correlation between psychological readiness to return to sport and athlete fear avoidance, r=−0.51 (95% CI −0.69 to −0.27), p<0.001. Therefore, lower fear is associated with a higher readiness.

### Differences in AFAQ responses between ‘ready’ and ‘not ready’ groups

There are differences present in the ‘ready’ and ‘not ready’ groups when answering the AFAQ, the results show two questions with a significant difference between the two groups. Those who were ‘not ready’ (M=2.0) believe that their current injury has jeopardised their future athletic abilities more than those who are ‘ready’ (M=1.5), p=0.040 (Question 5). Those who are ‘not ready’ (M=3.4) are less comfortable to return to playing until they are 100% as opposed to those who are ‘ready’ (M=2.6), p=0.037 (Question 6) (Figure 2).

## Discussion

The current study aimed to determine the influence of psychological readiness in athletes when returning to sport after injury and found that almost two-thirds (60%) of athletes were categorised as ‘not ready’ to return to sport after injury. This was in relation to a study by Glazer in 2009 ^[11]^, which found that I-PRRS scores were low after injury. They assessed the athletes at three other points in the study and showed increases in the I-PRRS scores as rehabilitation progressed before reaching a plateau before returning to competition. Evans et al. (2000) ^[13]^ had similar results in their study and also found that the I-PRRS scores were highest right before the athletes returned to sport.

An explanation for the differences in I-PRRS scores not being significant in our study is that the questionnaire was completed at different stages in their rehabilitation process, and specific injury time points had not been noted. Hence, the selected athletes ranged from recently injured athletes to those who had completed their rehabilitation process and were preparing to return to sport.

Another important finding was that the low confidence scores after injury were recorded by the athletes who spent more time away from their sport due to sustaining more severe injuries than those who did not. This belief was supported by a study which found that the time when athletes sustain a severe injury and then recover can be emotionally difficult. ^[14]^ In addition to the feeling of a loss of achievement of athletic potential, the loss of revenue can be financially overwhelming. ^[15]^ All these factors can have lingering psychological consequences, impacting not just the athletes’ journey to return to their sport but their likelihood of returning to sport at all. ^[14]^ However, an important consideration regarding the number of injuries sustained is that this study was conducted during the COVID-19 pandemic. This resulted in fewer competitions during this period, which may have led to fewer injuries sustained than would occur at a time of normal competition.

Additionally, this study aimed to determine the differences in reported fear avoidance behaviours in athletes who were or were not psychologically ready to return to play. The athletes were separated into two groups: those who were ‘ready’ and those who were ‘not ready’ to return to play, depending on their scores on the I-PRRS questionnaire. The results show that those in the ‘not ready’ group had marginally higher Athlete Fear Avoidance Questionnaire (AFAQ) scores than those in the ‘ready’ group. This shows that the athletes in the ‘not ready’ group were more afraid and apprehensive when returning to sport, than those in the ‘ready’ group.

Similarly, Monahan (2018) ^[16]^ also separated athletes into two groups of ‘ready’ and ‘not ready’ and found that the athletes in the ‘not ready’ group had significantly higher fear avoidance scores than those in the ‘ready’ group. In addition, the current study found that one of the questions which indicated a significant difference between the ‘ready’ and ‘not ready’ groups was that those who were ‘not ready’ believed that their injuries had jeopardised their future athletic abilities more than those who were ‘ready’.

Fischerauer et al. (2018) ^[17]^ found that athletes who are more apprehensive about returning to play have stronger fear avoidance behaviours. This may lead to destructive thoughts, which could deceive athletes into perceiving their injuries as being worse and more severe than they are.

A second objective which identified significant differences between the two groups found that those who were ‘not ready’ were less comfortable returning to play until they were 100% fit, compared to those who were ‘ready’. This is further explained in a study by Johnston and Carroll (1998), ^[18]^ who found that when athletes are injured, they are afraid to return to their sport unless they are back at 100% fitness, and re-injury anxiety can arise. This can lead to decreased performance upon return to sport due to constant awareness about situations that may replicate the mechanism of injury, and this can sometimes influence athletes to become very protective of the injured area. How this can occur is by athletes heavily strapping the injured area or by becoming hyper-aware of other sport-related conditions that could result in injury. This can, in turn, increase their risk of sustaining the same type of, or a different, injury in a different area. ^[18]^ Furthermore, previous literature has also shown that returning athletes to sport before they are psychologically ready can lead to fear, anxiety, and a decline in performance, ^[19]^ as the higher athletes scored in fear avoidance, the lower their physical function. ^[17]^

In contrast to athletes not wanting to return until their injury is 100% healed, Bianco (2001) ^[20]^ found that some athletes do not necessarily exhibit fear on this point, which is often driven by eagerness to get back to play. Reasons for this range from athletes either wanting to display physical superiority and trying to reduce their recovery time; ^[20]^ or feeling pressure to return to their position on their team as soon as possible due to feelings of insecurity about being replaced by someone else. ^[5]^ This, in turn, could also prompt athletes to return to sport much earlier than they should, before the rehabilitation process has effectively ended or before they have been medically cleared to return. This can possibly lead to an increased risk of re-injury and of not being able to perform at their pre-injury levels due to insufficient recovery. ^[20]^

It is recommended that if an athlete’s I-PRRS score is not high, defined as a score of 50 or above, waiting a little longer before returning the athlete may be the best option. ^[11]^ This does bring about an important consideration when looking at the results of the study because those athletes who have scored <50 on the I-PRRS could be returning to play too soon, especially when looking at the results of the AFAQ about this as these athletes are prone to one or more of the various fear avoidance behaviours manifesting either before they return to their sport or upon their return to play.

### Study Limitations

There is a need for further research in this area, with a larger sample size, for more representation and generalisability of the results. There should be a gender balance so that accurate observations can be inferred between male and female athletes. The differences in AFAQ and I-PRRS scores between genders, sporting codes, age groups, and types of training should be assessed. This will provide a more diverse baseline knowledge, affording sports medicine personnel, athletes, and coaches the ability to act effectively.

## Conclusion

Coaches and athletic trainers must be educated about the psychological effects of sustaining an injury, which can influence the athlete’s readiness to return to sport. In addition to this, they need to become familiar with how to use the tools available to assess athletes’ psychological states of mind.

Athletes’ psychological readiness to return to sport and their fear fluctuates at different times in the rehabilitation process.^[11]^ It is, therefore, vital to assess their psychological states of mind at different stages in the rehabilitation process. Ideally, there should be an increase in psychological readiness to return to play and a decrease in fear as the rehabilitation progresses. However, should this not happen, concerns can be identified and addressed timeously.

## Supplementary Information
