# Supplementary material for: The influence of psychological readiness of athletes when returning to sport after injury
Source: S Afr J Sports Med. 2024 Feb 15;36(1):v36i1a16356. doi: 10.17159/2078-516X/2024/v36i1a16356 (PMC10878413; doi:10.17159/2078-516X/2024/v36i1a16356)
Supplement: Supplementary file 1 [file 2078-516X-36-v36i1a16356-s001.pdf]

The influence of psychological readiness of athletes when returning to sport after injury

Supplementary Figures

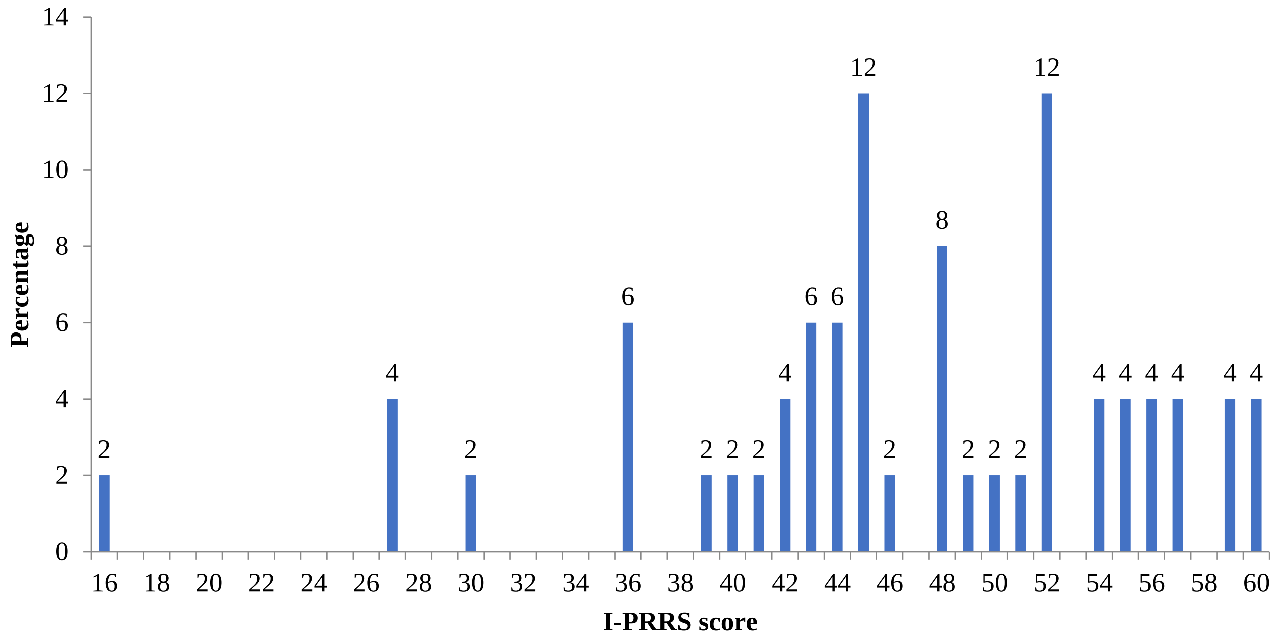

Fig S1. *Distribution of I-PRRS scores*

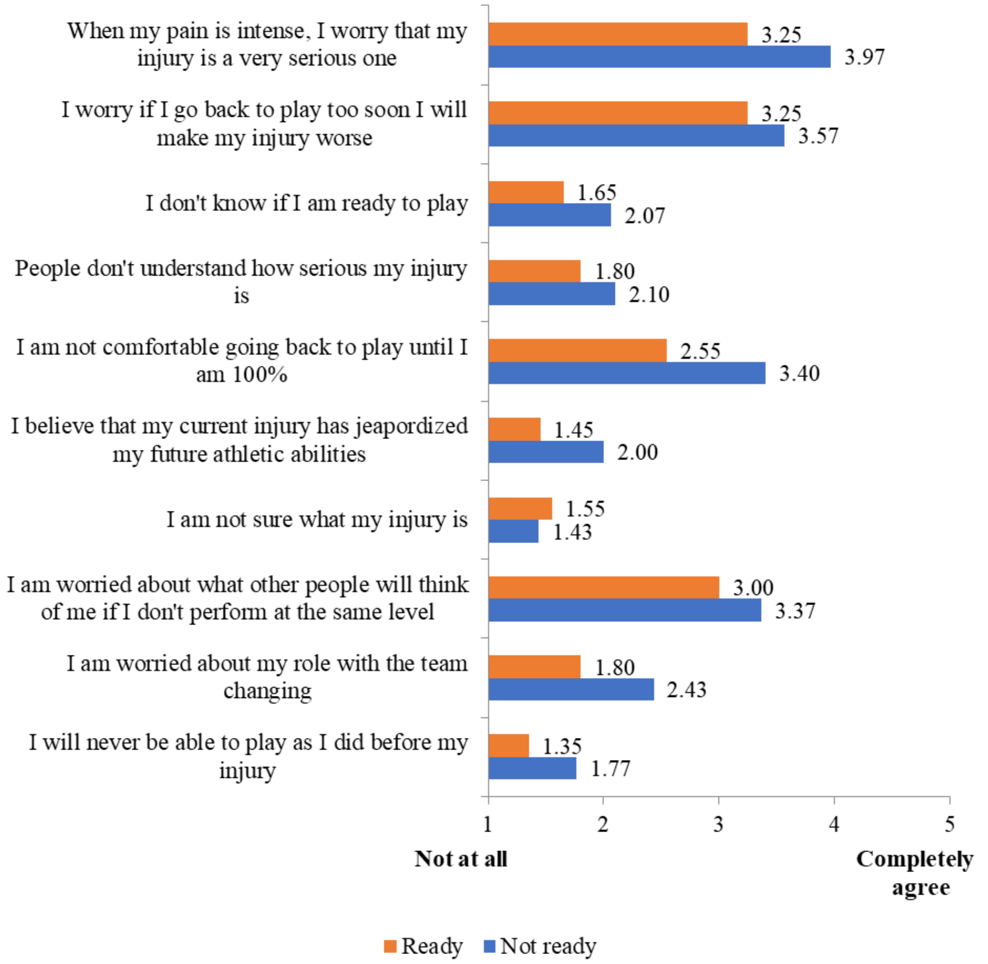

Fig S2. *Differences in mean scores between the two groups per AFAQ question*
